# Model of vitamin and mineral deficiency for toxicological research: Apoptosis activity under conditions of CCL4 intoxication

**DOI:** 10.1016/j.toxrep.2018.12.005

**Published:** 2018-12-18

**Authors:** Nadezhda V. Tyshko, Svetlana I. Shestakova

**Affiliations:** Federal State Budgetary Scientific Institution "Federal Research Centre of Nutrition, Biotechnology and Food Safety", Moscow, Russian Federation

**Keywords:** AIN, American Institute of Nutrition Rodent Diets, CCl4, carbon tetrachloride, LD50, median lethal dose, In vivo experiments, Biomarkers, Apoptosis, Wistar rats, CCl4, Vitamin deficiency, Mineral deficiency

## Abstract

The apoptosis activity and clinical state in vitamin and mineral supplemented male Wistar rats was evaluated after carbon tetrachloride exposure (CCL4). The animals were divided equally into 6 groups (3 control groups and 3 exposure groups) with the control groups (C-75, C-30, C-19) receiving AIN-93, a specific diet for rodents, consisting of a 75%, 30% and 19% ratio of vitamins (B1, B2, B3, B6) and minerals (Fe3+ and Mg2+) and exposure groups (E-75, E-30, E-19) receiving the same diet paradigm as with the control groups but with the additional CCL4 administered once a week as an olive oil solution (control groups received the same ratio of olive oil without CCL4) for a duration of 64 days.

The systemic condition of the male Wistar rats was evaluated based on morphological parameters and hematological and biochemical analysis, whereas the apoptosis activity in the liver was evaluated via comet assay techniques.

The apoptosis activity in the liver of control and exposure groups increased compared to the decrease in the essential substance provisions with the E-75 group reaching 129% (p < 0.05) higher levels compared to the C-75 group, and 98% (p < 0.05) and 23% (p > 0.05) higher in the E-30 and E-19 groups compared to the C-30 and C-19 groups, respectively.

From the apoptosis results and clinical state evaluation, it is clearly demonstrated that the effectiveness of using apoptosis activity as a biomarker after CCL4 exposure and the vitamin and mineral absorption capability in male Wistar rats can be applied as an evaluating method for toxicological research.

## Highlights

• The apoptosis activity in vitamin and mineral supplemented male Wistar rats was evaluated after carbon tetrachloride exposure (CCL4).
• The apoptosis activity in the liver of control and exposure groups showed an increase against a decrease in essential substances provision.
• Stress triggered the apoptosis activation through both nervous and humoral regulatory mechanisms.
• Groups with a maximum (75%) content of essential substances demonstrated a more pronounced result.

## Graphical abstract

**Figure:**
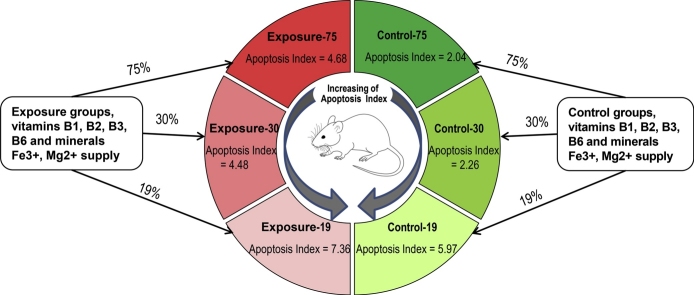

## 1 Introduction

Vitamins and micronutrients have been extensively studied showing that when a deficiency of an essential substance exists, it can play a major role in following associated metabolic disorders [1,2]. In particular, iron is a component of cytochromes which are involved in the metabolism of xenobiotics, as well as part of catalase, a key enzyme of the antioxidant defense system [3]. Magnesium, which is a part of more than 350 enzymes, regulates oxidative processes and effects the production of a number of cytokines and other humoral factors [4,5]. Vitamins in the B group, which are cofactors of oxidoreductases, provide energy to all biochemical processes in the body, including the processes of maintaining an antioxidant status [3,6]. Thus, taking into account just a few of the previously described examples of vitamin and micronutrient deficiency, it is important to discover new methods to clarify which exact parameters are to blame and how are they able to induce possible adverse effects.

One of the current trends in modern science is the research and development of toxicological methods, aiming to clarify hidden adverse effects of various low toxic chemical factors. Throughout the previous years, ways of obtaining such results was achieved by identifying new sensitive and specific biomarkers [[7], [8], [9], [10], [11]], choosing new biological objects (e.g., genetically modified organisms and organisms of synthetic biology that are highly similar to humans in their biochemical, physiological, pathological aspect of view) [[12], [13], [14]], running computer simulations [[15], [16], [17]] and by developing new toxicological models for laboratory animals [[18], [19], [20]].

Based on the above, the development of a toxicological model for laboratory animals, in this case exposed male Wistar rats, was preferred focusing on a modified diet composition in vitamins and minerals [21,22] and the relevant impact on their systemic condition after CCL4 exposure.

To confirm the efficiency of this model, a series of tests were conducted, fundamental to the adaptive potential of male Wistar rats, with emphasis on the immune status, antioxidant status, xenobiotic metabolism and lysosome membrane enzyme and apoptosis activity.

The purpose of this study was to establish the suitability of apoptosis activity indicators for use as possible biomarkers of toxic influence under conditions of reduced adaptation potential.

## 2 Materials and methods

### 2.1 Animals and administration protocol

One hundred and eighty male Wistar rats (weighing between 85.6 ± 1.0 g each) were used in this study. The animals were housed in plastic cages (2 per cage) with wood shavings in a steady heated (21 °C to 23 °C) air conditioned room with natural light settings. Modifications within the AIN-93 [23] diet administered, a newer formulated type diet created by the American Institute of Nutrition Rodent Diets and extensively used in in vivo experiments was provided ad libitum as well as drinking water, involving a gradual decrease in the content of B vitamins (thiamine, riboflavin, niacin and pyridoxine), iron and magnesium salts in the salt and vitamin mixtures (see Table 1). Rats were obtained from the Affiliated Unit "Stolbovaya" of Scientific Center for Biomedical Technology of the Federal Medical and Biological Agency. The animals were divided equally and randomly into 6 groups (3 control groups and 3 exposure groups) with the control groups (C-75, C-30, C-19) receiving AIN-93, a specific diet for rodents, consisting of a 75%, 30% and 19% ratio of vitamins (B1, B2, B3, B6) and minerals (Fe3+ and Mg2+) and exposure groups (E-75, E-30, E-19) receiving the same diet paradigm as with the control groups but with the additional CCL4 (≥99.5%, 289116 Sigma-Aldrich) administered once a week as an olive oil solution (control groups received the same ratio of olive oil without CCL4) with each injection containing 0.81 g/kg body weight of CCl4 that corresponded to 1/8 of the LD50 [24], see Table 2, reaching a total of 8 doses with each animal receiving a total of 6.5 g/kg body weight of CCl4 within the duration of the 64 day experimental study.

**Table 1:** Diet modifications.

Ingredient	Groups, % of essential substances supplying (as initial diet was used AIN-93 [23])		
	75%	30%	19%
**Vitamins, mg per kilogram feed**			
Thiamine (B1) CAS 67-03-8	3000	1200	760
Riboflavin (B2) CAS 83-88-5	2250	900	570
Niacin (B3) CAS 59-67-6	11300	4500	2850
Pyridoxine (B6) CAS 58-56-0	3750	1500	950
**Mineral substance, mg per kilogram feed**			
Magnesium oxide CAS 1309-48-4	630	250	160
Iron citric acid CAS 3522-50-7	160	60	40

**Table 2:** Study design.

Group	Notation of group	Diet	Content of vitamins B1,B2, B3 and B6, minerals Fe3+ and Mg2+ in the diet, % from basic AIN-93 level	Impact: weekly intraperitoneal injections, 8 in. per rat in total	Injectable substance	
					Olive oil	CCl_4_ olive oil solution
Control-75	C-75	AIN-93	75%	+	+	–
Exposure-75	E-75			+	–	+
Control-30	C-30		30%	+	+	–
Exposure-30	E-30			+	–	+
Control-19	C-19		19%	+	+	–
Exposure-19	E-19			+	–	+
Reference-75	R-75		75%	–	–	–
Reference-30	R-30		30%	–	–	–
Reference-19	R-19		19%	–	–	–

Throughout the study period all rats were regularly observed and their condition was closely monitored. Animals were examined based on appearance, movement and behavior patterns, skin and hair state, eyes and mucous membranes, excreta and respiration. Feed intake was measured every two days, body weight of rats was registered weekly, material sampling for investigations was done on the 64th day of the experiment.

The euthanasia procedure was completed after the animals were deprived of food for 12 h via physical method (decapitation) and all procedures performed were done according to the American Veterinary Medical Association (AVMA Guidelines for the Euthanasia of Animals: 2013 Edition) and Rules of laboratory practice approved by Order of the Ministry of Health of the Russian Federation No. 193n of 01/01/2016.

### 2.2 Comet assay technique

**Fig. 1:**
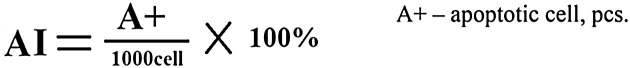
Apoptosis index formula.

The comet assay is an efficient method for obtaining details regarding DNA damage and possible repair procedures and is widely accepted in the field of in vivo research experiments, and is an official test validated by OECD [25]. The method of alkaline gel electrophoresis of isolated cells (DNA comets) [26] was used to assess the DNA fragmentation degree and to calculate the apoptosis index [[27], [28], [29]]. The electrophoresis was performed with a Sub-Cell GT Cell system (Bio-Rad Laboratories) and reagents used were from of Sigma Chemical Co. The apoptosis index (AI) was calculated by the formula shown in Fig. 1.

**Fig. 2:**
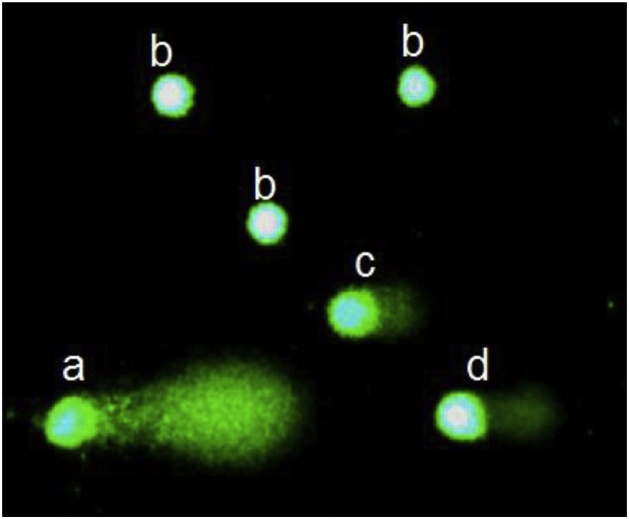
Types of rats liver cells. a ‒ apoptosis-positive cell (more than 30% of damaged DNA). b ‒ normal cells. c-d ‒ cells with different levels of DNA damage.

Via a provided constant electric field in the agarose gel, damaged DNA particles migrate to the anode and form a "comet's tail", hence the name of this method, where the length and size of the comet depends on the degree of inflicted DNA damage (Fig. 2). Microscopic analysis was performed on a Zeiss Axio Imager Z1 microscope at a magnification of 400 × . The images of DNA comets (SYBR Green I, S9430 Sigma-Aldrich) were analyzed using the software Comet Imager system, Metasystems GmbH. The percentage of DNA in the tail of comets (% of DNA in the tail) was used as an indicator of apoptotic cells. At least 100 cells were analyzed from each microsample. The results are represented as M ± m and min-max, where M stands for sample mean of measured data, m stands for the standard error, and min–max are the minimal and maximal values, as well as shares (percentage) or absolute figures.

Since intraperitoneal injections could pose a stress factor that could affect the apoptosis activity, the data analysis was carried out with taking into consideration the reference level of apoptosis (combined data from control animals, used in similarly designed experiments, groups R-75, R-30, R-19, respectively). In accordance with the experiment design, the quantitative and qualitative characteristics of the exposure groups were compared with the corresponding control and reference groups.

### 2.3 Statistical methods

Statistical analysis was completed with the use of SPSS 17.0 software package (IBM, USA) [30,31]. The homogeneity of variance and normal distribution between groups was determined by the chi-square test, while the variance equality was measured by the Student *t*-test method. Statistical significance was assigned at the P < 0.05 level.

## 3 Results

Throughout the experiment, there was no indication of systemic condition deterioration of the animals. The animals of controls and exposure groups didn’t show any differences in external appearance. The rate of growth depended on the administered vitamins with the initial bodyweight of all groups starting at 85.6 ± 1.0 g, and ending at 403.4 ± 8.7, 378.7 ± 8.1 and 338.7 ± 7.7 g for the C-75, C-30 and C-19 groups and 373.4 ± 8.3, 352.0 ± 7.0 and 326.9 ± 8.7 g for the exposed groups, E-75, E-30 and E-19 respectively. The noticeable difference between the groups reached 7% (E-75:C-75), 7% (E-30:C-30) and 4% (E-19:C-19) by the end of the study. Feed intake didn't differ between control and exposure groups, but only between groups with the varied essential substances levels, where the feed intake had a tendency to decrease (C-75→C-30→C-19 row) from 18 g to 20 g and 22 g, respectively.

The apoptosis activity in the liver of control and exposure groups showed an interesting result where when there was an increase in apoptosis activity, there was a decrease in the essential substances provision (Table 3). The apoptosis index in the C-75 group was 11% (p > 0.05) and 193% (p < 0.05) lower than in the C-30 and C-19 groups, E-75 and E-30 groups showed approximately the same levels, whereas the apoptosis index in the E-19 group was 57% higher (p < 0.05) than in the E-75 group.

**Table 3:** The apoptosis index of liver cells with CCl4 intoxication.

Apoptosis index, %	Groups		
	Reference value (stress free animals) N* = 3000	Control N = 1500	Exposure N = 1500
**Groups with 19% essential substances supplying**			
M ± m	1.58 ± 0.42	5.97 ± 1.01^1^	7.36 ± 0.51
*Min-Max*	*0.20-6.79*	*1.8-7.59*	*6.22-8.66*
**Groups with 30% essential substances supplying**			
M ± m	1.67 ± 0.52	2.26 ± 0.93	4.48 ± 0.40^2^
*Min-Max*	*0.08-5.81*	*0.79-4.82*	*3.44-5.60*
**Groups with 75% essential substances supplying**			
M ± m	2.01 ± 0.32	2.04 ± 0.97	4.68 ± 0.65^2^
*Min-Max*	*0.39-3.58*	*0.35-5.60*	*3.26-6.86*

N*- the total number of analyzed cells in each group.

1 Differences with background values are significant at p < 0.05.

2 Differences with control are significant at p < 0.05.

There were no statistically significant differences noticed between the R-75, R-30 and R-19 groups, but the apoptosis level tended to decrease in a R-75→R-30→R-19 row. R-75 rats had an apoptosis index of 17% and 21% (p > 0.05) higher than those of R-30 and R-19 rats. Data analysis revealed that the apoptosis activity in all control groups was higher than reference values with differences were found between the C-75 and R-75, C-30 and R-30, C-19 and R-19 groups by 2% (p > 0,05), 35% (p > 0.05), and 277% (p < 0.05), respectively (Table 3). Apoptosis activity in E-75 group appeared to be 129% (p < 0.05) higher than in C-75 group and 98% (p < 0.05) and 23% (p > 0.05) higher in E-30 and E-19 groups against C-30 and C-19 groups, respectively.

## 4 Discussion

As seen in the presented data, when the absence of a stress factor exists, the rats with a reduced intake of vitamins B1, B2, B3 and B6 and minerals (Fe3+ and Mg2+) demonstrate a slight decrease regarding apoptosis activity. This is probably due to overexpression of the anti-apoptotic Bcl-2 protein genes initiated by vitamin deficiency [32]. The apoptosis activity analysis from the control and reference groups, which differed only in receiving or not intraperitoneal injections, suggests that stress triggered the apoptosis activation in animals of all control groups through both nervous and humoral regulatory mechanisms. As a result of this stress system activation, the cell membranes were depolarized by nerve impulses, forcing to open potential-dependent calcium channels and consequent delivery of extracellular Ca2+ to the cell. In the cytoplasm, Ca2+ links to the intracellular calmodulin receptor and activates calmodulin-dependent protein kinase, which initiates glycolysis mobilization, inhibits glycogen re-synthesis, and increases ATP and oxygen consumption, thus providing the stress adaptation [[33], [34], [35]].

As a natural physiological antagonist of calcium ions, deficient Mg2+ in a diet deteriorates the intracellular Ca2+/Mg2+ balance and leads to the predominance of Ca2+ activating Ca2+-sensitive proteases and lipases, and damaging then the membranes. The Mg2+ deficiency formation in rats was confirmed by the results of the biochemical studies: Mg2+ content in the blood serum of control and exposure groups was ˜25%, 15% and 8% below the lower limit of the normal under conditions of 19%, 30% and 75% essential substances consumption, respectively. Since the groups did not differ in the level of stress exposure, the apoptosis activity growth against the essential substances reduction accounts for a growing Ca2+/Mg2+ imbalance in the first place. This hypothesis was supported by a apoptosis activity decrease in C-19→C-30→C-75 row, whereas the reference groups demonstrated a totally different trend, which assumes the prevailing stress and micro-element imbalance influence over the activity of anti-apoptotic Bcl-2 proteins.

Apoptosis activity growth under stress exposure and essential substances reduction could also be caused by a general decrease in the adaptive potential, that leads, among other things, to intensifying free radical lipid oxidation, and contributes to the apoptosis activation [36,37].

Apoptosis enhancement in exposure groups with exposure to CCl4 intoxication can be explained by the very nature of this toxicant metabolism by accumulating in the liver as a lipophilic hepatotropic compound, causing fatty degeneration and centrolobular necrosis. The damaging effect of CCl4 is associated with the development of oxidative stress, where in severe cases can lead to destruction of intracellular mitochondrial and lysosome membranes, release of hydrolytic lysosomal enzymes, protein denaturation and cell death [[38], [39], [40]]. It should be noted that CCl4 exposure enhanced the apoptosis activity, however a scale of differences between exposure and corresponding control groups was decreasing from 75% to 30% to 19%, an indication of a limited maximal apoptosis level in the organism. The regulation of programmed cell death processes is quite complex, has multiple stages and requires a considerable energy consumption. Therefore, in cases of overwhelming exposures, it might demand a lower need of resources for the organism to activate the necrosis mechanisms in order to shed the damaged cells [37,38].

Groups with a maximum (75%) content of essential substances demonstrated a more pronounced result, hence the importance of monitoring apoptosis activity indicators in regards to in vivo toxicological experiments when an optimal supply of vitamins and minerals are provided is proven. Furthermore, the stress from the regular intraperitoneal injections raised an apoptosis level in the liver, with a highest increase in rats suffering from an essential substances deficiency. In this case one of the principle mechanisms of apoptosis activation included a growing Ca2+/Mg2+ imbalance and disruption in the oxidation reactions regulation, intensifying the generation of free radicals [40,41]. Consequently, the apoptosis indicators can't be considered as a useful tool for studies involving stress exposures so as to prevent overestimated results.

## 5 Conclusions

The results of the present study clearly demonstrate the effectiveness of using apoptosis activity as a biomarker in accordance with the experiment design, animal nutrition, active substance level and toxicant administration method. This indicator may be most effective if combined with analyzing the activity of xenobiotics metabolism, lysosome and antioxidant system enzymes, and lipid peroxidation processes.

## Conflict of interest statement

The authors states that there are no conflicts of interest.

## Funding

The research was conducted with the financial support of the Russian Science Foundation (grant No. 16-16-00124).
